# Vitamin K for kidney transplant organ recipients: investigating vessel stiffness (ViKTORIES): study rationale and protocol of a randomised controlled trial

**DOI:** 10.1136/openhrt-2019-001070

**Published:** 2020-07-15

**Authors:** Jennifer Susan Lees, Kenneth Mangion, Elaine Rutherford, Miles D Witham, Rosemary Woodward, Giles Roditi, Tracey Hopkins, Katriona Brooksbank, Alan G Jardine, Patrick B Mark

**Affiliations:** 1 Institute of Cardiovascular and Medical Sciences, University of Glasgow, Glasgow, UK; 2 Renal Medicine, NHS Greater Glasgow and Clyde, Glasgow, UK; 3 AGE Research Group, NIHR Newcastle Biomedical Research Centre, Newcastle University, Newcastle upon Tyne, UK; 4 Queen Elizabeth University Hospital, NHS Greater Glasgow and Clyde, Glasgow, UK

**Keywords:** risk factors, CT scanning, MRI, clinical trials, renal disease

## Abstract

**Background:**

Renal transplant recipients (RTRs) exhibit increased vascular stiffness and calcification; these parameters are associated with increased cardiovascular risk. Activity of endogenous calcification inhibitors such as matrix gla protein (MGP) is dependent on vitamin K. RTRs commonly have subclinical vitamin K deficiency. The Vitamin K in kidney Transplant Organ Recipients: Investigating vEssel Stiffness (ViKTORIES) study assesses whether vitamin K supplementation reduces vascular stiffness and calcification in a diverse population of RTR.

**Methods and analysis:**

ViKTORIES (ISRCTN22012044) is a single-centre, phase II, parallel-group, randomised, double-blind, placebo-controlled trial of the effect of vitamin K supplementation in 90 prevalent RTR. Participants are eligible if they have a functioning renal transplant for >1 year. Those on warfarin, with atrial fibrillation, estimated glomerular filtration rate <15 mL/min/1.73 m^2^ or contraindications to MRI are excluded. Treatment is with vitamin K (menadiol diphosphate) 5 mg three times per week for 1 year or matching placebo. All participants have primary and secondary endpoint measures at 0 and 12 months. The primary endpoint is ascending aortic distensibility on cardiac MR imaging. Secondary endpoints include vascular calcification (coronary artery calcium score by CT), cardiac structure and function on MR, carotid-femoral pulse wave velocity, serum uncarboxylated MGP, transplant function, proteinuria and quality of life. The study is powered to detect 1.0×10^–3^ mm Hg^-1^ improvement in ascending aortic distensibility in the vitamin K group relative to placebo at 12 months. Analyses will be conducted as between-group differences at 12 months by intention to treat.

**Discussion:**

This trial may identify a novel, inexpensive and low-risk treatment to improve surrogate markers of cardiovascular risk in RTR.

## Background

Cardiovascular disease (CVD) is the leading cause of premature mortality in patients with renal disease.^1^ For patients with end-stage kidney disease (ESKD), renal transplantation affords the greatest benefit in terms of CVD risk reduction compared with dialysis, but renal transplant recipients (RTR) remain at 3–4 times elevated CVD risk compared with the general population^2^ and die prematurely from CVD, often with a functioning kidney transplant.^3^ Among patients with advanced chronic kidney disease (CKD), vascular stiffness and calcification are common,^4^ linked and associated with cardiovascular events and mortality.^5–7^ Standard secondary prevention therapies—such as maintenance blood pressure targets and cholesterol-lowering agents—are recommended in the transplant population,^8^ however, these strategies are inadequate in RTR with non-traditional risk factors for CVD.^9 10^

The development and progression of vascular stiffness and calcification are heavily influenced by the activity of calcification inhibitors,^11^ such as matrix gla protein (MGP), osteocalcin and Gla-rich protein.^12^ These proteins are reliant on conversion to their active forms by a vitamin K-dependent post-translational modification (gamma carboxylation). Among RTR, vitamin K intake is inadequate^13^ and subclinical deficiency is prevalent and associated with CVD and mortality.^14^ By optimising the function of vitamin K-dependent calcification inhibitors, vitamin K supplementation may offer a cost-effective and low-risk treatment option to reduce long-term cardiovascular risk and mortality and maximise organ preservation in RTR. By testing vitamin K supplements at supraphysiological dose, we may nullify the confounder that patients with diet rich in vitamin K are ‘healthier’ overall, with lower CVD risk and better outcomes.^15^

Trials of vitamin K to improve vascular stiffness and calcification show promising results in various populations.^16^ We designed the Vitamin K in kidney Transplant Organ Recipients: Investigating vEssel Stiffness (ViKTORIES trial) to investigate the hypothesis that vitamin K supplementation improves vascular stiffness and calcification over 1 year in prevalent RTR.

## Methods and analysis

### Trial design and participants

**Figure 1:**
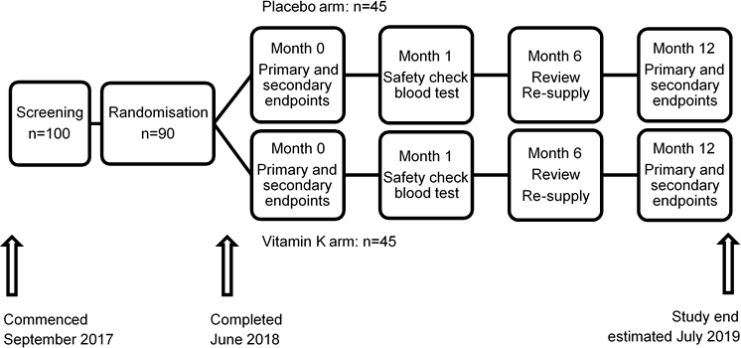
ViKTORIES study design and timetable. ViKTORIES, Vitamin K in kidney Transplant Organ Recipients: Investigating vEssel Stiffness.

The ViKTORIES trial is a single-centre, phase II, parallel-group, randomised, double-blind, placebo-controlled trial in prevalent RTRs (figure 1). We enrol adult participants (18 years or over with a functioning renal transplant implanted >12 months and with estimated glomerular filtration rate (eGFR—measured by the chronic kidney disease epidemiology collaboration (CKD-EPI) equation^17^) >15 mL/min/1.73 m^2^. Patients are excluded if they meet any of the following criteria: known permanent or paroxysmal atrial fibrillation, concurrent warfarin use, taking vitamin K or indication for vitamin K, allergy to gelatine, lactose or cellulose (constituent ingredients of the capsules or packing materials used to make the interventions), breast feeding or of childbearing potential, known glucose-6-phosphate dehydrogenase (G6PD) deficiency, life expectancy <12 months, standard contraindications to MRI^18^ or inability to provide written informed consent in English.

### Interventions

Menadiol diphosphate is a synthetic, water-soluble form of vitamin K, licensed for use in the UK for treatment of haemorrhage associated with deficiency of vitamin K-dependent clotting factors. This form of vitamin K is likely to be better absorbed than standard fat-soluble forms in those with malabsorption syndromes. Using this form of vitamin K facilitates production of a matching placebo, in contrast to other licensed, liquid forms of vitamin K (phylloquinone).

Interventions—either 5 mg menadiol diphosphate or matching placebo—are administered orally three times per week (Monday, Wednesday and Friday) for 1 year. This dose of menadiol diphosphate was selected to ensure excess vitamin K was present for use as cofactor for vitamin K-dependent carboxylation. Size 4 gelatine capsules are prepared containing menadiol diphosphate 5 mg and packed with microcrystalline cellulose Ph. Eur. (Tayside Pharmaceuticals, Dundee, UK). Identical capsules are packed with microcrystalline cellulose Ph. Eur. as placebo.

Menadiol diphosphate was originally sourced from licensed 10 mg tablets (Alliance Pharmaceuticals, Chippenham, UK) which were crushed and packed into capsules at the desired 5 mg dose. Due to a supply issue of licensed menadiol tablets after study commencement, capsules are now manufactured using 5 mg menadiol diphosphate United States Pharmacopeia (USP) with lactose as packing material but all other manufacturing the same. No interruption to dose or supply of dispensed study medication occurred due to this change of preparation.

### Randomisation sequence generation

A computer-generated code list is provided by Sealed Envelope (Sealed Envelope, London, UK) in a password-protected file to drug manufacturer (Tayside Pharmaceuticals) to produce identical, sequentially numbered bottles containing either vitamin K or placebo. The code list is organised in random permuted blocks to facilitate unstratified 1:1 allocation ratio.

### Allocation concealment mechanism

Randomisation is conducted by study investigators or trained research nurses using a custom-built, password-protected online system, created and maintained by Sealed Envelope.

### Implementation

Local investigators (JSL, ER, PBM and AGJ) recruit participants from routine outpatient transplant clinic appointments in National Health Service (NHS) Greater Glasgow and Clyde (Scotland, UK) and enrol participants into the study, including obtaining informed consent and randomising according to methods detailed above.

### Blinding

Local investigators, research nurses, pharmacy staff and participants are blinded to treatment allocation by use of numbered but otherwise identical medication bottles. Investigations including laboratory blood and urine samples, pulse wave velocity measurements, quality of life questionnaires are conducted by blinded study investigators or laboratory staff. After enrolment in the study, participants are given a 5-digit study ID (2-digit site code ‘01’ followed by 3-digit sequential screening code).

### Data capture

A custom-designed electronic case report form is used, designed (by JSL) using Castor Electronic Data Capture (www.castoredc.com; Amsterdam, Netherlands).

### Outcomes

#### Primary outcome

The primary outcome is between-group difference in ascending aortic distensibility at 12 months measured by analysis of covariance (ANCOVA), adjusting for covariates of age, duration of ESKD and the baseline value of ascending aortic distensibility. Measured by cardiac MRI, aortic distensibility is an accurate and highly reproducible measure of aortic stiffness^19^ and is an early predictor of CVD and mortality.^20–22^

#### Secondary outcomes

All secondary outcomes are assessed as between-group differences at 12 months by ANCOVA, treating age, duration of ESKD and the baseline value of the outcome of interest as covariates. Secondary outcome measures include the following: coronary artery calcification score by non-contrast CT, carotid-femoral pulse wave velocity and augmentation index (SphygmoCor XCEL PWA and PWV software, AtCor Medical, Australia), MR measures of cardiac structure and function (descending aortic distensibility, left/right ventricular mass, function and strain, T1 and T2 relaxation times), markers of vitamin K status: desphospho-uncarboxylated MGP (dp-ucMGP), office blood pressure, ECG, bone metabolism and turnover (calcium, phosphate, parathyroid hormone, 25-hydroxyvitamin D), transplant function, proteinuria and quality of life (EuroQol-5 Dimension-5 level instrument^23^). Several other biomarkers will be tested as secondary outcomes if funding allows: calcification propensity (T50), elastin degradation products, bone metabolism and turnover (1-25hydrovitamin D, fibroblast growth factor-23, osteocalcin, fetuin, bone morphogenetic protein, tartrate-resistant acid phosphatase-5b), cardiovascular markers (high-sensitivity troponin, brain natriuretic peptide) and endothelial function (asymmetric dimethylarginine). Additional plasma, serum and urine are collected and frozen for future testing of unspecified biomarkers (with consent), which may include but are not limited to biomarkers of vitamin K status (other than dp-ucMGP) and tubulointerstitial fibrosis. Participants are asked to keep a food diary for 28 days for estimation of dietary vitamin K content.

### MR acquisition technique

**Figure 2:**
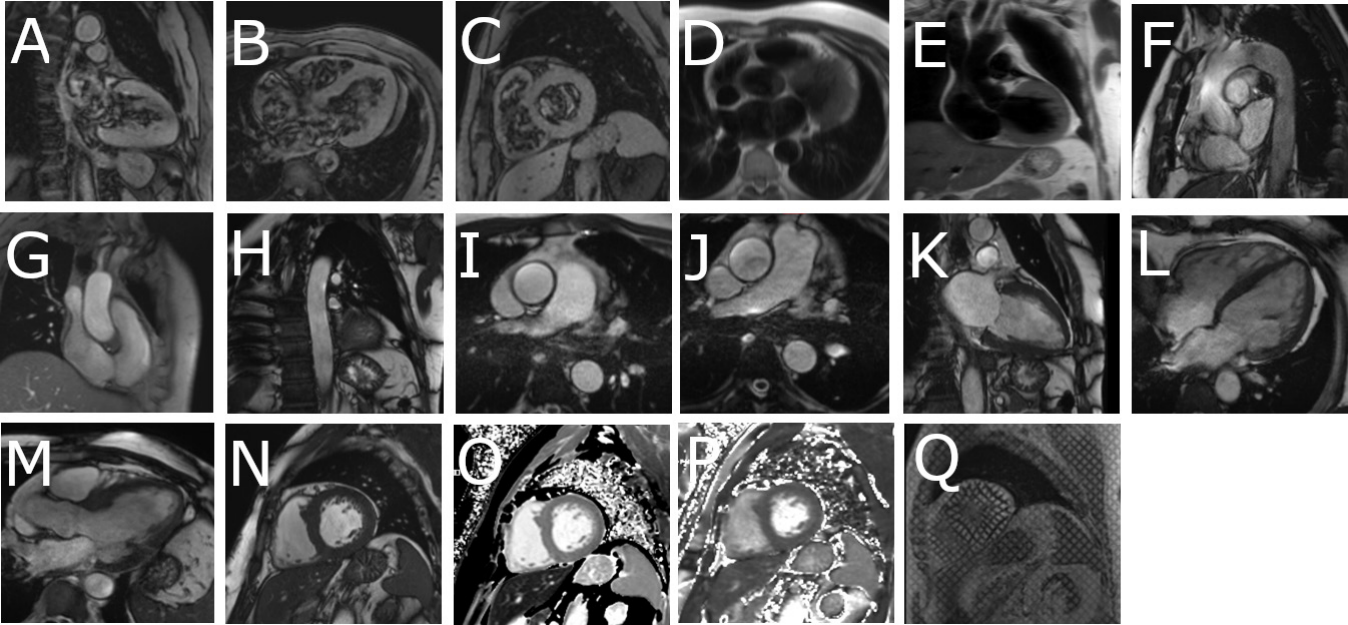
Illustrative images of MRI scan acquisition. (A) Two-chamber localiser; (B) four-chamber localiser; (C) left ventricular short axis localiser; (D) haste in transverse plane; (E) haste in coronal plane; (F) aorta cine; (G) orthogonal ascending aorta; (H) orthogonal descending aorta; (I) transverse cine ascending aorta; (J) transverse cine descending aorta; (K) vertical long axis cine; (L) horizontal long axis cine; (M) left ventricular outflow tract cine; (N) left ventricular short axis stack cine; (O) T1 map mid segment; (P) T2 map mid segment; (Q) left ventricular short axis tagging mid segment.

Participants undergo non-contrast cardiac MR on a Siemens Prisma 3T scanner (Siemens Inc, Erlangen, Germany) in the research MR facility at the Queen Elizabeth University Hospital, Glasgow. An 18-channel body array is used anteriorly with a 32-channel spine array for posterior acquisition. Scans are gated by electrocardiography. Typical image acquisition sequence is summarised in table 1. Illustrative images of selected MRI sequences are displayed in figure 2.

**Table 1:** Typical MRI acquisition sequence

Scan type	Region	MRI sequence type	Time
**Localiser**	Thorax	Three plane localisers	1 min
	Cardiac	Two and four-chamber views Short axis localiser	1 min
**Anatomical**	Thorax	Haste in transverse and coronal planes	4 min
**Aorta**	Aorta cine	TrueFISP/gradient echo cine	1 min
	Orthogonal ascending aorta	TrueFISP/gradient echo cine	1 min
	Orthogonal descending aorta	TrueFISP/gradient echo cine	1 min
	Transverse cine and flow ascending aorta	TrueFISP cine and flow	5 min
	Transverse cine and flow descending aorta	TrueFISP cine and flow	5 min
**Functional cardiac**	Horizontal long axis Vertical long axis Left ventricular outflow tract	TrueFISP cine 7 mm thick TrueFISP cine 7 mm thick TrueFISP cine 7 mm thick	4 min 4 min 4 min
	Short axis stack (left ventricle)	TrueFISP cine 7 mm thick/3 mm gap	10 min
**Myocardium**	Cardiac (limited)	T1 Map x 3 (short axis: basal, mid, apical)	2 min
		T2 Map x 3 (short axis: basal, mid, apical)	0.5 min
		Tagging (short axis image: mid segment)	0.5 min
**Total time**			**44** min

Cine imaging of the ascending and descending aorta is acquired for measurement of vessel size for distensibility measurements, at the level of the right pulmonary artery for the ascending aorta and approximately 10 cm above the diaphragm for the descending aorta. Typical imaging parameters are: field of view (FOV) 340×286 mm, slice thickness 7 mm, TR −20.7 ms, TE 1.51 ms, flip angle 50°, voxel size 1.33×1.33x7 mm.

Left ventricular (LV) mass and function are assessed from balanced steady state free precession cine imaging (TrueFISP) in long axis planes (vertical long axis, horizontal long axis and LV outflow tract) then with sequential short axis LV cine images (7 mm slice thickness, 3 mm gap between slices) from the atrioventricular ring to the apex. Typical scan parameters are as follows for retrospectively gated TrueFISP 2d (tfi2d) sequence: FOV 340×286 mm, slice thickness 7 mm with 3 mm gap in short axis (SA) stack, repetition time (TR) – 41.4 ms, echo time (TE) 1.51 ms, flip angle 50°, voxel size 1.33×1.33 x 7 mm, 30 calculated phases.

Parametric maps are acquired from three short-axis images at the basal, mid and apical segments of the left ventricle and tagging from a single short-axis image at the mid segment of the left ventricle. A T2 prepared sequence, BEAT map turbo flash 2d, was acquired with the following typical imaging parameters: FOV 360×288 mm, slice thickness 8 mm, TR—207 ms, TE 1.3 ms, flip angle 12°, voxel size 1.9×1.9 x 8 mm. T2 preparations x3 are imaged over a 9-heartbeat period with duration 0 ms, 30 ms, 55 ms and with a 3-heartbeat recovery period.

A motion-corrected 2D BEAT map sequence (tfi2d) optimised for heart rate is used to acquire T1 maps. Typical imaging parameters are: FOV 360×306 mm, slice thickness 8 mm, TR—280 ms, TE 1.12 ms, flip angle 35°, TI 180 ms, voxel size 1.4×1.4 x 8mm. Two T1 preparations are imaged over an 11-heartbeat period with a starting T1 time of 100 ms with increment of 80 ms.

### MR image assessment

MR images are analysed for primary and secondary endpoints as above using Argus (Siemens Inc, Erlangen, Germany) and Cvi42 (Circle Cardiovascular Imaging, Calgary AB, Canada) software by an investigator blinded to treatment allocation (JSL).

### Aortic distensibility analysis

**Figure 3:**
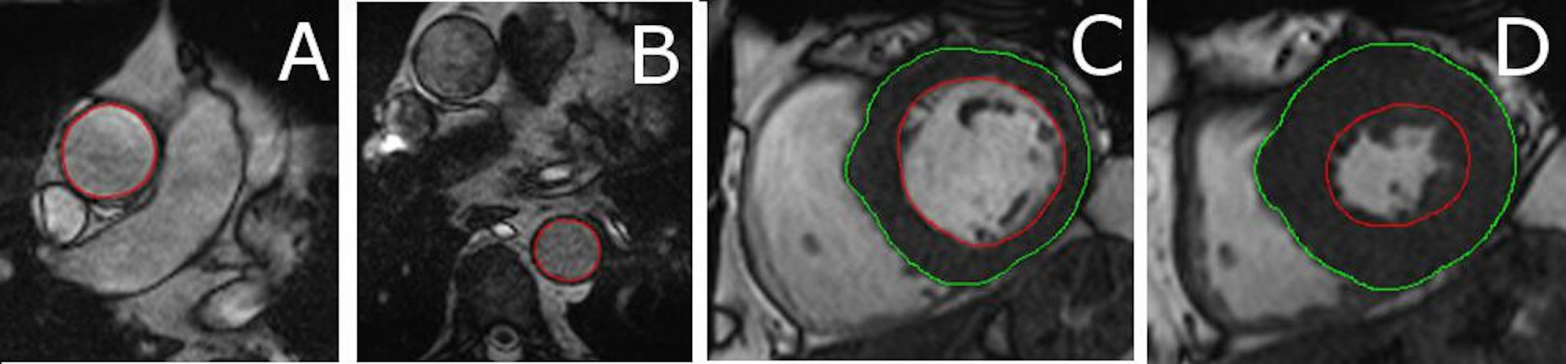
A and B: contours drawn around the endovascular border of the ascending aorta (A) for estimation of ascending aortic distensibility and descending aorta (B) for assessment of descending aortic distensibility. C and D: endocardial (red) and epicardial (green) borders outlined at end-diastole (C) and end-systole (D) on short axis cine images to estimate left ventricular mass and function. Papillary muscles excluded from left ventricular mass measurements.

Aortic volumes are measured by manually tracing the endovascular border of the ascending and descending aorta (figure 3A/B). Non-invasive brachial blood pressure is recorded simultaneously with capture of cine images using a non-ferromagnetic cuff (Philips Expression MR400 and Expression IP5, MRI Devices, Knaresborough, UK), and aortic distensibility (mm Hg^-1^) in ascending and descending aorta calculated from the following equation^21 24 25^:

AD=[Maximum aortic volume (mL)]–[Minimum aortic volume (mL)]

[Minimum aortic volume (mL)]xPulse Pressure (mm Hg)

### Left/right ventricular mass and function analysis

Endocardial and epicardial borders are outlined at end-diastole and end-systole on short axis cine images to estimate LV mass and function in standard fashion^26^ (figure 3C/D). Using body weight and height acquired immediately before scanning, the end-systolic and end-diastolic volumes and LV mass are indexed to body surface area.

### Strain

Endocardial and epicardial contours are defined as above on LV short-axis views, and inferior reference point is defined at the insertion point of the right ventricle. LV and RV extent, endocardial and epicardial contours are defined at end-diastole and end-systole on vertical long axis and horizontal long axis views. Three-dimension (3D) left and right ventricular strain analysis is conducted using dedicated feature-tracking software.

### Parametric map analysis

LV contours are defined on raw T1 and T2 images then copied onto the colour-enhanced spatially coregistered maps. The anterior right ventricular-septal insertion point is used as a reference, and both T1 and T2 maps analysed for 16 segments according to the American Heart Association (AHA) model (apex segment 17 excluded).^27^ User-defined semiautomated border delineation is used to create AHA regions of interest of similar size and shape, taking care not to overlap with other tissue interfaces. Relaxation times are measured in each of the 16 segments.

Acceptable segments are identified by assessing individual segments for susceptibility and motion artefacts. Those segments affected by artefact are removed and a mean global T1 or T2 time calculated from the remaining segments. An average septal T1 or T2 time is calculated by using acceptable anteroseptal, inferoseptal and septal AHA segments (segment numbers 2, 3, 8, 9 and 14).

### Intra and interobserver variability

There will be blinded reanalysis of n=20 randomly selected datasets, in random order, for intraobserver and interobserver variability for all primary and secondary MR outcomes above.

### CT Image acquisition

Images for coronary artery calcification score are obtained using an Aquilion ONE Vision Edition CT scanner (Canon Medical Systems, Crawley, UK) in the Clinical Research Imaging Facility at the Queen Elizabeth University Hospital in Glasgow. Scans are acquired with a single heartbeat non-contrast volumetric scan; adaptive iterative dose reduction (AIDR) 3D iterative reconstruction is employed for radiation dose minimisation.

### CT image assessment

**Figure 4:**
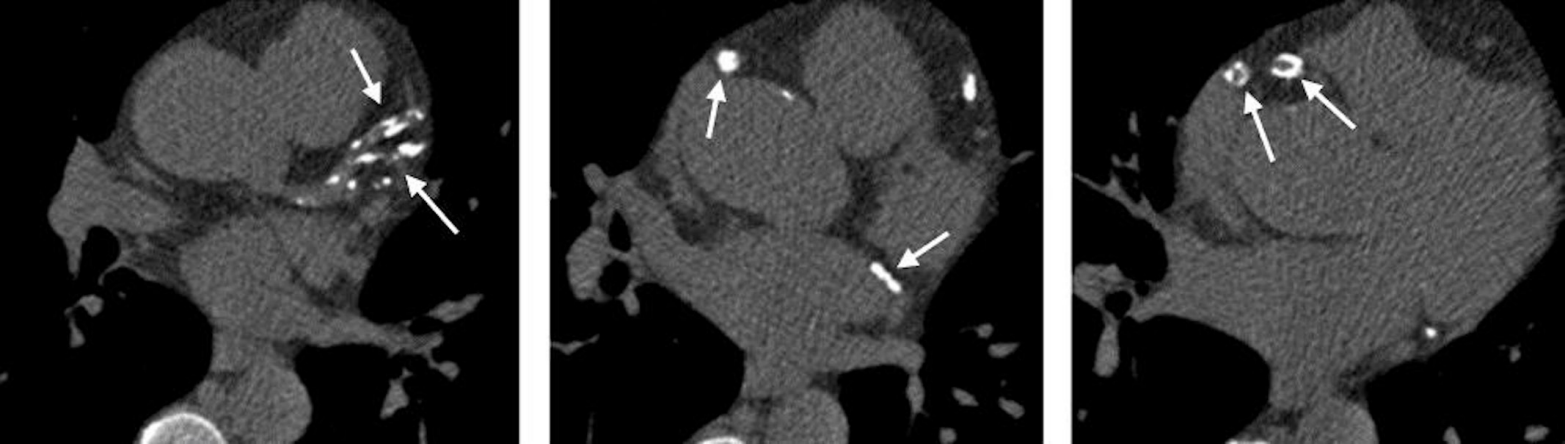
Cross-sectional, non-contrast CT images of the heart. Total coronary calcification score is calculated for each artery according to the density and area of calcification (arrows) observed in each artery. The sum of calcification scores in each artery gives a total coronary calcium score.

Coronary artery calcification (Agatston) score^28^ is calculated using Vitrea Advanced Visualisation software (Vital Images Inc, Minnetonka, Minnesota, USA) by a consultant radiologist with 14 years experience blinded to treatment allocation (GHR), excluding segments with previous coronary artery stents in situ (figure 4).

### Statistical considerations

The ViKTORIES study has a statistical analysis plan in line with Consolidated Standards of Reporting Trials (CONSORT) guidelines. We will analyse differences between treatment and placebo groups by intention to treat as the primary analysis, and will conduct per-protocol analyses as sensitivity analyses. Analysis of the primary outcome (between-group difference in ascending aortic distensibility at 12 months) will be performed by ANCOVA adjusting for age, duration of ESKD and baseline values. Secondary analyses will similarly be by between-group ANCOVA, adjusting for age, duration of ESKD and baseline values of the variable of interest. All prespecified secondary outcomes will be reported in study publications. Subgroup analyses will be performed in older versus younger participants (age >65 vs <65 years) by analysing for heterogeneity of treatment effect. As a sensitivity analysis, we will perform multiple imputation for missing data for the primary outcome if the assumption of Missing at Random is met. The number and characteristics of serious adverse events (SAE) will be summarised as a whole and by study arm. Analyses will be conducted using R statistical software package (R V.3.5.3 or higher).

### Sample size

Sample size has been determined to detect a 1.0×10^–3^ (SD 1.3×10^–3^) mm Hg^-1^ improvement in ascending aortic distensibility in the intervention group relative to placebo at 12 months: a difference that we have previously shown to be associated with clinical difference in survival to death or cardiovascular event at 2 years.^21^ To achieve a power of 90% with alpha=0.05 requires 37 patients per group (74 in total). We assume a dropout rate of 20% over 12 months of follow-up (a conservative estimate based on previous studies) and aim to recruit 90 participants in total.

### Follow-up and timetable

The study timetable is illustrated in figure 1. Recruitment commenced in September 2017 and all 90 participants were recruited and randomised by end June 2018. The study will complete when the last participant completes the last follow-up visit (estimated by July 2019). Results are expected by December 2019.

### Registration

ViKTORIES was prospectively registered with ISRCTN Registry on 26/09/2017 (ISRCTN22012044).

### Ethics

The study is being conducted in accordance with the World Medical Association Declaration of Helsinki (1964) and its revisions. The trial was approved by the West of Scotland Research Ethics Committee 4 (Ref: 17/WS/0101 on 22/06/2017) and is sponsored by NHS Greater Glasgow and Clyde Research and Development Department (Ref: GN16RE696 on 22/09/2017). The study follows standard operating procedures of the trial Sponsor (https://www.glasgowctu.org/sops.aspx; NHS Greater Glasgow and Clyde, Scotland, UK as part of Glasgow Clinical Trials Unit). Trial progress is monitored by Sponsor representatives from pharmacy, research and development and by the Clinical Trials Manager (KB); routine audit was conducted by Sponsor in 2018. An annual report is issued to the Research Ethics Committee and to the funder (Kidney Research UK). The study follows CONSORT guidelines (http://www.consort-statement.org).

### Safety considerations

Menadiol diphosphate has few undesirable effects but allergic reactions have been known to occur rarely. Haemolytic anaemia has occurred rarely and is more common in G6PD deficiency; we exclude patients with this disorder. Similarly, we exclude patients with allergies to any constituent components of the intervention, including gelatine (found in capsules), lactose and cellulose (used as packing materials with menadiol diphosphate and as placebo). Patients will have monitoring of renal transplant function and immunosuppression levels in addition to standard care. All adverse events are recorded from the time a participant consents to join the study until the last study visit. Participants in this trial are expected to have multiple comorbidities and high levels of illness. The following are therefore classed as expected within this cohort of patients: death or hospitalisation due to new cardiovascular event, new diagnosis or treatment of cancer, fall or fracture, infection, exacerbation of an existing medical condition, deteriorating renal function, high or low potassium levels, nausea, vomiting, constipation or diarrhoea; admission for elective or planned investigation or treatment. For the purposes of this study, only suspected unexpected serious adverse reactions are collected in an expedited manner and reported to the sponsor via the pharmacovigilance office (within 24 hours of the site becoming aware of the event). These events are determined if they are suspected to be caused by the intervention (ie, an unexpected SAE that occurred after the initiation of the intervention and without a clear alternative explanation) but the events are not in keeping with information known about menadiol diphosphate. Participants with unresolved adverse events at the last study visit will be followed up until resolution or 30 days (whichever is sooner).

### Dissemination plan

Complete trial data will be submitted for presentation at an international meeting and will be published in a peer-reviewed journal, and shared on social media. After publication, ViKTORIES participants will be informed of trials results by letter and the dataset will be contributed to the Virtual International Renal and Transplant Trials Archive (www.virtualtrialsarchives.org/virtta).
